# Assessment of transmural perfusion effect with Blood Oxygen Level-Dependent Cardiovascular Magnetic Resonance Imaging (BOLD-CMR)

**DOI:** 10.1186/1532-429X-11-S1-P287

**Published:** 2009-01-28

**Authors:** Jacqueline Flewitt, Matthias Vöhringer, Jordin Green, Matthias Friedrich

**Affiliations:** 1 Stephenson CMR Centre, Calgary, AB Canada; 2 Robert-Bosch-Krankenhaus, Stuttgart, Germany; 3 Seimens Healthcare Canada, Calgary, AB Canada

**Keywords:** Cardiovascular Magnetic Resonance, Coronary Artery Disease Patient, Coronary Stenosis, Improve Image Quality, Suspected Coronary Artery Disease

## Background

OLD-CMR has successfully been used for the diagnosis of perfusion deficits caused by coronary stenosis. New BOLD-CMR sequences show improved image quality for assessing myocardial oxygenation with excellent spatial resolution, offering a distinct advantage over other image modalities. We hypothesized that these new BOLD-CMR techniques can detect differences in oxygenation throughout the myocardial wall.

## Objective

To investigate the epicardial and endocardial oxygenation with a new BOLD-CMR sequence in healthy volunteers compared to patients with suspected coronary artery disease (CAD).

## Methods

11 healthy volunteers (mean age 29 ± 4 years) and 11 patients with suspected CAD (mean age 59 ± 8 years) who all had abnormal perfusion results on SPECT were recruited for scanning. Using a clinical 1.5 T MRI system (MAGNETOM Avanto, Siemens Healthcare, Erlangen, Germany), SSFP BOLD-CMR was performed on a mid left ventricular short axis slice at baseline and during adenosine infusion (140 micro-g/kg). Typical scan parameters were: Field-of view 193 × 280 mm; matrix size 106 × 192; slice thickness 10 mm; T_R_/T_E_ 5.8/2.9 ms; flip angle 90°; typical breath-hold duration 14 s. Images were analyzed using clinically validated software (cmr^42^, Circle Cardiovascular Imaging Inc., Calgary, Canada). After automatic definition of the subendocardial 50% and the subepicardial 50% of the wall thickness, the BOLD signal intensity (SI) for each was analyzed and the relative change from baseline during adenosine infusion was calculated. Late enhancement was performed to ensure that patients did not have an infarct in the selected slice for analysis.

## Results

**Figure 1:**
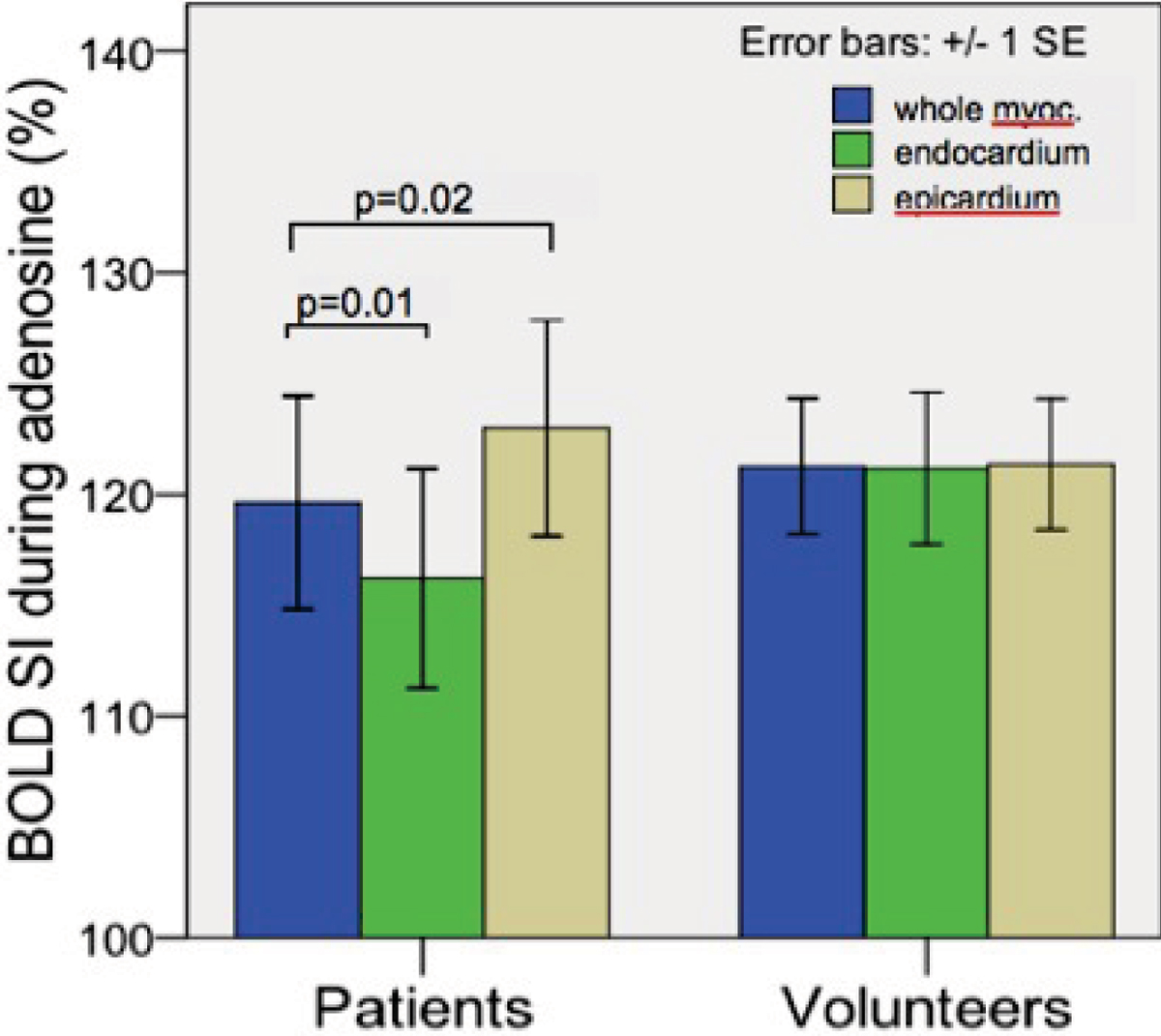
**BOLD SI changes for suspected CAD patients and healthy volunteers**.

Image quality was excellent in 8 patients and all healthy volunteers. Three patients were excluded due to motion artifacts on the stress portion of the exam. Results are illustrated in Figure 1 and summarized in Table 1 as means and standard error (SE). Heart rate (HR) increased from baseline with the infusion of adenosine. The mean HR increase was 48 ± 8% for the suspected CAD patients, and 29 ± 8% for the healthy volunteers. The increase in BOLD-SI of the whole myocardium from baseline to adenosine was similar in CAD patients (19.6 ± 4.8%) and in healthy volunteers (21.2 ± 3.0%). In healthy volunteers there was only a marginal difference between the BOLD-SI increase in the endocardium only, and epicardium only (mean difference 0.2 ± 1.6%, p = 0.88). However, in the suspected CAD patients, the endocardial BOLD-SI increases were significantly lower than epicardial BOLD-SI increases (mean difference 5.7 ± 2.2%, p = 0.03).

**Table 1:** Adenosine induced changes in BOLD SI and HR

	SI rel. to baseline whole myocardium		SI rel. to baseline endocardium		SI rel. to baseline epicardium		HR rel. to baseline	
	%	SE (%)	%	SE (%)	%	SE (%)	%	SE (%)
Patients with suspected CAD (n = 8)	119.6	4.8	116.2*	5.0	123.0*	4.9	148	8
Healthy volunteers (n = 11)	121.2	3.0	121.3	3.2	121.0	2.9	129	8

*p < 0.05 compared to whole myocardium.

## Conclusion

A state-of-the-art BOLD-sensitive MRI sequence, with its improved image quality and excellent spatial resolution, can detect differences in epicardial and endocardial myocardial oxygenation and are feasible for the clinical setting.

